# Eight-Element Communication Model for Internet Health Rumors: A New Exploration of Lasswell’s “5W Communication Model”

**DOI:** 10.3390/healthcare10122507

**Published:** 2022-12-11

**Authors:** Haibin Wei, Jianyang Chen, Xinyan Gan, Zhenyi Liang

**Affiliations:** 1 Public Management College, Guangxi University, Nanning 530004, China; 2 Public Health and Management College, Guangxi University of Chinese Medicine, Nanning 530200, China

**Keywords:** Internet health rumors, 5W communication model, grounded theory

## Abstract

(1) Background: Rumors are a special type of information. Based on the classic theory of the communication of information, the “5W” communication model, this article aims to build a new model and thus explains the generation and communication of Internet health rumors. (2) Methods: The authors selected 50 Internet health rumors, which were widely spread in widely used websites and social media in China, then grounded theory is used to perform the qualitative analysis of the Internet health rumors. (3) Results: Three Core Concepts are abstracted after qualitative analysis. An internal dynamic mutual assistance mechanism of the communication of rumors is built and illustrated. Based on Lasswell’s “5W” communication model, the authors develop an eight-element communication model for Internet health rumors to illustrate the generation and communication of Internet health rumors. (4) Conclusions: By removing one or several elements of this new model, the chain of the communication of Internet health rumors could be cut off, which is valuable information for the government or websites to manage communication of Internet health rumors.

## 1. Introduction

We are surrounded by health rumors. When a pandemic rages, health rumors spread even more rapidly. The COVID-19 pandemic is the most recent example. Compared with traditional rumors, Internet rumors are characterized by faster spreading, wider influence, greater social harm, higher professionalism, greater concealment, and larger temptation. The Internet is gradually becoming an important platform for people to gain health information that is easily made up and spread. Against the backdrop of the “Healthy China” strategy, the governance of Internet health rumors has become one of the difficult problems faced by the Chinese government. The Chinese government has strengthened the governance of Internet rumors, but there are many problems with rumor control.

This study focuses on the micro-level of Internet health rumors to profoundly explore the flexibility and applicability of the 5W communication model based on three questions, “How are Internet health rumors generated?” “How are Internet health rumors spread?”, and “How do elements of the generation and communication of Internet health rumors interact with each other?” A new theoretical communication model is constructed to answer the questions about Internet health rumors.

## 2. Literature Review

As stated in Webster’s Dictionary of the English Language, rumor refers to gossip, talk, or opinion without a discernible source or known authority for its truth. With rumors in Massachusetts as the case, Knapp initially studied the causes, consequences, and control strategies of rumors [1]. The process of rumor spreading was concisely described by formulas and models by several scholars. Allport believes that rumor = importance (event) × fuzziness (event), pointing out the direct proportion between the occurrence of rumor and the importance and fuzziness of events [2]. Chorus improved this formula into rumor = importance (event) ×fuzziness (event) × critical ability of the public, believing that rumor spreading is correlated with the public’s critical ability [3]. Other models of communication of rumors are also proposed.

Among the Internet rumors, health rumors are the worst. False information spreads significantly farther and faster than true information does [4]. Health misinformation also applies to this rule. For example, misinformation about the Zika virus was three times more likely to be shared than verified stories on social media sites [5]. The reason is that health misinformation was novel and resulted in more emotions such as disgust, fear, and surprise [4] (p.2). Scholars proposed specific ways to reduce the spread of health misinformation. For example, rumor-dispelling warnings such as “this tweet may contain misinformation” would decrease users’ likelihood of sharing a health rumor [6]. Additionally, the corrective health information posted by experts or authoritative institutions on social media can reduce the dissemination of health misinformation effectively. [7]

The 5W communication model provides a theoretical basis for explaining the propagation mechanism of Internet health rumors. The spread of Internet health rumors is a subdivision of information dissemination. When it comes to information dissemination, it is necessary to mention the well-known “5W communication model”. American sociologist Harold Lasswell officially put forward the “5W model” in his book *The Structure and Function of Communication in Society* [8]. The “5W” refers to Who, What, In Which Channel, To Whom, and With What Effect. There are different attitudes towards the contribution of Lasswell and his 5W model of communication, which has triggered heated debates in the academia. Shoemaker, Tankard, and Lasorsa consider that the “5W model” was one of the earliest and most influential communication models [9]. Biagi (2013) considers it to be one of the primary drivers behind current conceptions of mass communication [10]. However, McQuail believes it to be conceptually dated due to its linear orientation [11]. Especially after the emergence of the Internet, considering the impact of the Internet on the 5W model, the scholar maintains that the study on communication should break through the bottleneck of linear structure and be promoted to a perspective of media and social relations [12].

To sum up, those who support the 5W model believe that the 5W model effectively illustrates the process and elements of information dissemination in society, and the model still has its explanatory power and applicability even to this day. The opposing view is that the 5W model is outdated and unable to adapt to the environment of the Internet, but few researchers have put forward any improvement plans. This study holds that we should dialectically explore new value and application of the 5W model rather than simply support or oppose it. For instance, by expanding the five elements, Peng found that “Under new media environment, communication process and the research of such five elements will have greater opportunities and broader prospects for development [13].” We quite agree with the view of Sapienza who believes that Lasswell’s construct is inherently flexible enough to meet the theoretical needs of today’s scholars [14]. On this basis, the process and elements of information dissemination described by the 5W model can provide a theoretical basis and analytical framework for this study, and the 5W model is also believed to be applicable and expandable at the micro level of Internet health rumors.

## 3. Materials and Methods

### 3.1. Sources of Text Materials

We collected 50 Internet health rumors which are widely spread as text materials. Institutions for science communication, social media, news websites, and government would release the lists for the most popular Internet rumors in the field of health and science—for example, China Association for Science and Technology, WeChat, Sina website, Toutiao website, and Beijing News, etc. The topics are usually about food safety, medicine safety, and environment issues. The rumors appeared from 2014 to 2019. We selected the classic Internet health rumors from these lists. These rumors are suitable to serve as empirical data. According to the generation mechanism of these text materials, it is possible to explain the mechanism and pattern of generation and dissemination of Internet rumors.

The information for 50 Internet health rumors is in Appendix A, including title of rumor, name of the lists, the platforms, and website addresses.

### 3.2. Coding Program

#### 3.2.1. Coding Procedure

This qualitative study is based on text materials. With the coding technology of the grounded theory, the Nvivo12.0 software is used to encode the data step by step, until it reaches theoretical saturation when there is no new concept. The grounded theory requires researchers to carry out two parts of work. (1) Open coding is the initial stage of constant comparative analysis, before delimiting the coding to a core category and its properties [15]. (2) Selective coding means that “the analyst delimits coding to only those variables that relate to the core variable in sufficiently significant ways to be used in a parsimonious theory” [16].

In this study, the coding technology of the grounded theory is applied in two aspects. First of all, open coding is conducted by labeling the content related to the basic theme in the text materials and recording the feelings after reading the text materials. Secondly, selective coding is to sort and classify the labels, refine the key factors of the generation and dissemination of Internet health rumors, and summarize the internal relationships among the key factors.

#### 3.2.2. Reliability and Validity

A unified standard improves the validity of qualitative research. The research team formulated a detailed coding guideline through discussion and selected two analysts to code separately by following the coding guideline. The cross-validation shows that 90% of the nodes coded by the two analysts are consistent. Code comparison verifies the reliability of the qualitative study. The coding nodes achieved full coverage of the 50 Internet health rumors from 2014 to 2019, and no new nodes appeared, which indicated high validity of the coding results.

#### 3.2.3. Results of Coding of Text Materials

After open coding, the text materials are summarized as 55 Primary Concepts (see Table 1). The grounded theory requires the researchers to conduct selective coding to generalize the Primary Concepts to Core Concepts. During the process of the first selective coding, the team classified the Primary Concepts into nine Category Concepts (See Table 2). For the second selective coding, nine Category Concepts are generalized into three Core Concepts, that is, Correlation, Credibility, and Communication (See Table 3).

## 4. Discussion

### 4.1. Generation of Three Core Concepts and Their Relationship

#### 4.1.1. Generation Based on Correlation and Credibility

**Figure 1:**
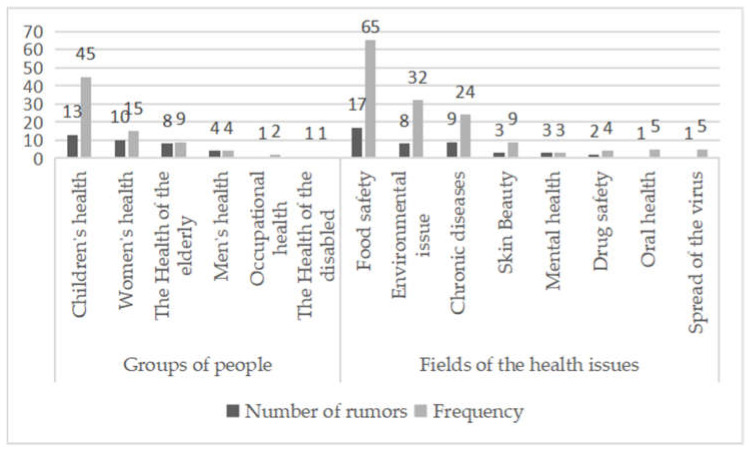
Number of rumors based on groups of people and encoded frequency based on fields of Internet health rumor.

“Correlation” is the prerequisite for the generation of Internet health rumors. The public’s actual demands for health information have led to the generation of Internet health rumors. Based on the results of open coding, regarding the groups of people that are targeted, Internet health rumors related to children’s health are responded to most frequently, with such rumors being encoded 45 times among the 13 text materials. Regarding the fields of the health issue involved, the highest response frequency was found in Internet health rumors about food safety, with such rumors being encoded 65 times among the 17 text materials (Figure 1). The frequent occurrence of Internet health rumors concerning children’s health and food safety is associated with the psychology of caring for children by parents and the concern about the food. In addition, when fabricating a fictitious person or place in a vivid context, Internet health rumors are becoming seemingly authentic. Hence, Internet health rumors have been closely related to the health issues of different groups of people. Hence, the rumors attract different groups of people to read and focus on.

“Credibility” guarantees the generation of Internet health rumors. The Category Concept “Faking real information” is a common trick for generating Internet health rumors, the Category Concept “fuzzy truth” is a necessary condition for generating Internet health rumors, and the Category Concept “Claiming as reliable information” makes Internet health rumors more mysterious. As early as 1947, the generation of rumors was proportional to the importance and fuzziness of events. Credibility directly determines the “quality” of Internet health rumors.

#### 4.1.2. Communication by Duplication and Variation

The ultimate goal of rumor mongers is to spread Internet health rumors. From the selective coding results, the Core Concept “Communication” involved 49 text materials and is encoded 713 times as the most frequently encoded core category. Moreover, according to the coding frequency of 50 text materials in the core category matrix, except for “Claiming as reliable information”, Core Concepts “Correlation” and “Credibility” highly frequently respond to the concept “Communication” as shown in Table 4.

The spread of Internet health rumors mainly refers to duplication and variation. First of all, duplication needs to attract the attention of the public, with the eye-catching headline being the most representative approach. Secondly, it is necessary to meet psychology of the public. When people are anxious about topics related to themselves, they create and spread rumors [17]. Fear of cancer and curiosity are the main psychological motivation for the public to spread Internet health rumors. Thirdly, to extensively spread Internet health rumors, secondary transmission is induced. It is manifested in different ways. For example, the Primary Concept of “moral kidnapping” means the health rumors are connected it to the receivers’ families (e.g., “Just spread it out, simply for the health of your family”). Videos’ commentary makes rumors incendiary. Taking advantage of rumor’s derivatives is the way that using another rumor to verify the original rumor which makes them more credible. Economic benefits are used as the bait to spread the rumors (e.g., rumor mongers distribute a small amount of money in a chatting room in “WeChat” to stimulate spreading). The key factor “acquaintances” induce the varied communication of Internet health rumors. Rumors do not come from strangers, but from our acquaintances [18]. For example, the function “Moments” of social media is the best place for posting articles, pictures and videos. Internet health rumors are shared and posted there and are easily shared by other friends with a simple click. The spread of Internet health rumors is intensified by the promotion of “acquaintances”. Even health issues that originally have little to do with people become closer to life simply due to the recommendation, comments, or spread by acquaintances. They are particularly able to stimulate, trigger, or arouse people’s attention even if it is only browsing [19].

#### 4.1.3. Internal Dynamic Mutual Assistance Mechanism

**Figure 2:**
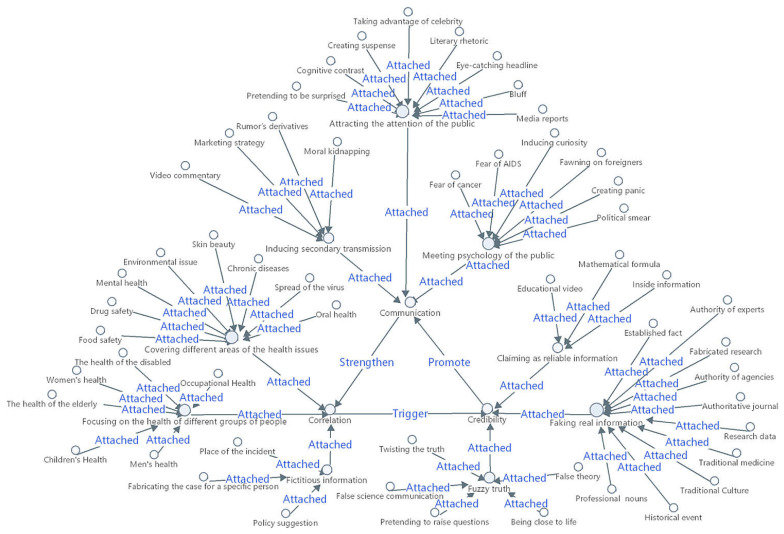
Internal dynamic mutual assistance mechanism (relationship among Correlation, Credibility, and Communication).

Correlation, Credibility, and Communication are the three key factors of the generation and communication of Internet health rumors. Correlation, as the source power, triggers Credibility if people have great demands for health information but insufficient knowledge about health. Credibility promotes communication. Studies have shown that high credibility increases trust and the intention to share rumors [20]. Credibility makes rumors more persuasive, especially for Internet users who lack the motivation to process information [21]. Moreover, communication also affects credibility. According to the spiral of silence theory, when people holding certain opinions are in the “minority”, they tend to remain silent so as not to be isolated [22]. In the Internet environment, people who hold minority opinions tend to remain silent or even join the majority to protect themselves from cyber violence. Consequently, recurrent rumors are reinforced and become credible, because people do not spread rumors that they do not believe. They only believe what they choose to believe. Spreading dreadful rumors verify their prejudices to share feelings of fear and reduce discomfort [23]. Therefore, when rumors are spread to a certain degree, their correlation and credibility will be strengthened, as shown in Table 4. Figure 2 shows the relationship among Correlation, Credibility, and Communication and the process of the formation of Core Concepts from Primary Concepts.

### 4.2. Eight-Element Communication Model for Internet Health Rumors

The Internet environment diversifies the elements of the generation and communication of Internet health rumors. Internet health rumors cannot exist independently in Internet environment, and their generation and communication are deeply influenced by the external environment, especially in the era of “new media”.

**Figure 3:**
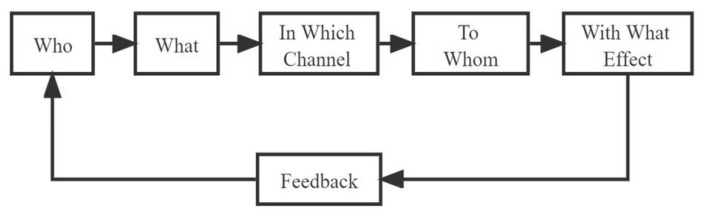
“5W” communication model that was proposed by Harold Lasswell.

The 5W communication model provides the theoretical basis for the communication of Internet health rumors. Harold Lasswell proposed the “5W” communication model in his book *The Structure and Function of Communication in Society*. “5W” represents “Who”, “What”, “In Which Channel”, “To Whom”, and “With What Effect” (Figure 3). The 5W communication model has the value of application and the room for extension. The application to the area of Internet health rumors is worth exploring.

“Who” is expanded to rumor mongers and refuters, and “What” is enlarged to Internet health rumor information, rumor-dispelling information, and hot events. To release their stress and tension [24] and take revenge on the society [25], rumor mongers make up Internet health rumors. The Internet environment is thereby filled with health rumors. Internet rumors severely endanger national political security, social stability, economic security, and social order [26]. Therefore, the government, authoritative media, and other rumor refuters do not allow the generation and communication of Internet health rumors.

Rumor refuters and mongers fight against each other, and rumor-dispelling information released by rumor refuters compete with Internet health rumors on the Internet. For example, the original text of Internet health rumors in some of the 50 samples are hardly found on the Internet because these rumors are dispelled by the government, scientific research institutions, authoritative media, and social media platforms. For these rumors, they are seen in the text of rumor-dispelling information as objects of analysis. Therefore, only rumor-dispelling information is shown to the public and the communication chain of the rumors is cut off. Thus, rumor refuter and rumor-dispelling information as new elements are necessarily added in the new communication model.

Hot events are likely to induce the public to emotionally spread Internet rumor information based on their prior experience [27]. Hot events have a weighted effect on the generation and communication of Internet health rumors. In this study, Internet health rumors which are widely spread are caused by hot events related to food safety, drug safety, disease, environment, and psychology. The hot events are closed to quality of life, health, and life, so it easily triggers the emotion of the public and leads to virus-like dissemination. For instance, due to the frequent occurrence of hot events related to food safety, the Internet health rumor that “spotted eggs are poisonous” in 2018 was highly similar to a short video popular in 2015. Thus, hot events is brought into the new communication model as a new element.

“In Which Channel” refers to the diversification of media channels. In the Internet environment, the media that spread Internet health rumors include websites, post-bars, BBS, Weibo, blogs, WeChat, instant messaging, and e-mail in diversified forms ranging from text to picture, sound, and video. Concerning the 50 Internet health rumors, they are from websites and social media, and the content are shown by texts, pictures, and short videos. It is more difficult to prevent Internet health rumors from spreading in multiple forms and via multiple media channels. Moreover, the evolution of media has not only affected the way of information transmission between people but also have brought a new impact on the “special information”, rumors.

“To Whom” indicates the diversification of receivers. The elderly, children, and women are the receivers accurately targeted by Internet health rumors. The Analysis Report of Population Vulnerable to Rumors released by Tencent Company in 2017 also shows that the elderly are most vulnerable to the spread of Internet rumors, and health rumors account for the highest proportion of rumors spread among the elderly (The report is released by Tencent Truth Platform in 2017). In addition, mutual confirmation between receivers is another factor that promotes the extensive spread of rumors. Apart from arousing the attention of receivers, Internet health rumors also have to make themselves convincing among the targeted receivers to achieve the purpose of communication. As pointed out by Kapferer, there are three conditions for rumors to be convincing: a) the source of information is reliable, b) the information should be what people expect and want to know, and c) the information should sound real [28]. The Category Concepts such as “faking real information”, “fuzzy truth”, “claiming as reliable information”, and “meeting the psychology of the public” in the coded materials exactly reveal the above viewpoints.

“With What Effect” is the effect or result of the generation and communication of Internet health rumors. A specific scenario is that the receiver reads (or does not read), believes (or does not believe), and spreads (or does not spread) the Internet health rumors. Based on the result of this study, Core Concepts are abstracted, namely, Correlation, Credibility, and Communication. Whether people read, believe, and spread an Internet health rumor is based on the three Core Concepts. These three Core Concepts constitute an internal dynamic mutual assistance mechanism for the generation and communication of Internet health rumors, which explains the generation and communication of Internet health rumors.

Therefore, Rumor Mongers, Rumor Refuters, Internet Health Rumors, Rumor-Dispelling Information, Hot Events, Media Channels, Receivers, and Effect constitute the eight elements throughout the generation and communication of Internet health rumors as shown in Table 5. Among them, rumor refuters, rumor-dispelling information, and hot events are three new elements added based on the 5W communication model; “Who”, ”What” “In Which Channel”, “To Whom” and “With What Effect” have also been given new meanings in the context of the Internet environment.

### 4.3. Relationships among Eight-Element Communication Model

**Figure 4:**
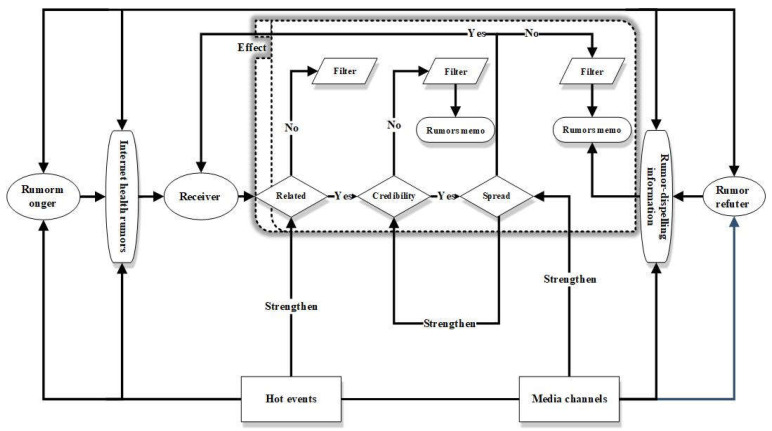
Relationship among eight-element communication model. Rumor memo: meaning the receiver knows that this health information is a rumor, marks it as “rumor”, and would not believe and spread it.

Coupling refers to the phenomenon that two or more systems or two forms of motion influence each other and even unite through interaction [29]. The key to coupling is to break the boundaries of the original system and recombine the related elements according to the natural association of the elements and the free flow of information and form a “living” subject system with self-organizing structure and dynamic elements in the system [30]. Based on the 5W communication model, we proposed the eight elements of the generation and communication of Internet health rumors. The coupling of Internet health rumors refers to the interaction and unification of rumor mongers, rumor refuters, rumor information, rumor-dispelling information, hot events, media channels, receivers, and effects through various ways and forms during the generation and communication of Internet health rumors. From different perspectives, the eight elements of the generation and communication of Internet health rumors have different combination forms to explain the process and mechanism of the generation and communication of Internet health rumors. The relationships are shown in Figure 4.

From the perspective of the system boundary, the eight-element communication model includes internal and external coupling. The internal coupling refers to the internal dynamic mutual assistance mechanism mentioned above among the three elements, namely correlation, reliability, and communication. Internet health rumors are “time bombs”. Once rumors are contacted by the receivers, the internal dynamic mutual assistance mechanism of Internet health rumors starts to work, and the receivers make the following choices: (a) unrelated → filter, (b) related → unreliable → filter → rumors memo, (c) related → reliable → not to spread → filter → rumors memo, and (d) related → reliable → spread → related. External coupling refers to how the seven elements interact with each other and with an internal mutual dynamic assistance mechanism. Whether people read (or do not read), believe (or do not believe), and spread (or do not spread) is determined by correlation, credibility, and communication, respectively. Therefore, the eight-element communication model is illustrated as follows: (a) rumor mongers → Internet health rumors → receivers → not to read, (b) rumor mongers → Internet health rumors → receivers → read → not to believe ← rumor-dispelling information ← rumor refuters, (c) rumor mongers → Internet health rumors → receivers → read → believe → not to spread ← rumor-dispelling information ← rumor refuters, and (d) rumor mongers → Internet health rumors → receivers → read → believe → spread ← rumor-dispelling information ← rumor refuters. The relationships are shown in Figure 4.

From the perspective of the development process, the eight-element coupling model includes the prelude to coupling, the occurrence of coupling, and removing coupling. Prelude to coupling refers to the stage in which the Internet health rumors made up by rumor mongers have not reached the receivers, that is, rumor mongers → Internet health rumors. At this stage, there is no coupling effect among the eight elements. The coupling effect is imminent because they aim at specific populations such as kids, women, or elders. Once rumors reach the receivers, the coupling effect occurs. The occurrence of coupling is the stage in which the Internet health rumors made up by rumor mongers have reached the receivers and produced certain effects, that is, rumor mongers → Internet health rumors → receivers → effect. The coupling effect is manifested as reading, believing, and spreading. According to the influence of the coupling effect, rumor refuters fight against rumor mongers and Internet health rumors by releasing them at different degrees. Removing coupling refers to the rumor-dispelling information. From the perspective of development, the three stages of the eight-element coupling model can be summarized and simplified as rumor mongers → Internet health rumors → receivers → effect ← rumor-dispelling information ← rumor refuters (Figure 4).

From the perspective of the number of elements, the eight-element coupling model includes weak coupling, regular coupling, and strong coupling. Weak coupling refers to the coupling phenomenon with incomplete main elements (rumor mongers, receivers, and rumor refuters) and the incomplete relationship between elements without intervention elements (hot events and media channels), e.g., rumor mongers → Internet health rumors → receivers → effect. Regular coupling refers to the coupling phenomenon with complete main elements (rumor mongers, receivers, and rumor refuters) and complete relationship between elements but without intervention elements (hot events and media channels), e.g., rumor mongers → Internet health rumors → receivers → effect ← rumor-dispelling information ← rumor refuters. Strong coupling refers to the coupling phenomenon with complete main elements (rumor mongers, receivers, and rumor refuters), the complete relationship between elements, and intervention elements (hot events and media channels), as shown in Figure 4.

In summary, the relationships among this eight-element communication model from three perspectives are illustrated. Based on this, the research team proposed the idea that the approach to the governance of Internet health rumors is to break the relationship among the eight elements.

## 5. Strategies for Governing Internet Health Rumors

### 5.1. From the Perspective of Rumor Mongers and the Channels: Controlling the Publishing and Spreading of Internet Health Rumors

Information source determines the origin of rumors, and also becomes a key factor in evaluating the credibility of Internet health rumors, which has a significant impact on the popularity and duration of Internet rumors of public events. The professionalism of the rumor monger and the authority of the platform for Internet health rumors directly affect whether the Internet health rumors would be read, believed, and spread. Therefore, only by tracking the information source and taking targeted measures can the Internet health rumors be effectively eliminated or reduced. At the same time, the government should pay close attention to hot issues in the health field to avoid or reduce the generating and spreading of health rumors on the Internet. For example, new Internet health rumors have surged since 2020, such as COVID-19, related medicine, and related vaccine. Regarding channels for communication of Internet health rumors, news media, social media, and other platforms should be hold accountable if they have no policy or actions for controlling Internet health rumors. The spread of some Internet health rumors could be cut off by those channels. The government should strictly monitor and control the channels and media for releasing and disseminating Internet health information, establish a regular governance mechanism, and conduct emergency management for the possible wrong and misleading public Internet health information.

### 5.2. From the Perspective of Rumor Refuter: Improving the Governance Ability of Internet Health Rumors

The government often plays the key role of “rumor refuter” in rumor control, and the credibility of the government is the key to effectively dispel rumors. If the government’s credibility is vulnerable, it may fall into the “Tacitus trap” (Tacitus trap: a political theory named after Roman historian Tacitus, which describes a situation where an unpopular government is hated no matter what it does and whether it is right or wrong), in the process of releasing refuting rumors on the Internet. In the field of health information communication, the credibility of the government is mainly reflected in the timeliness and transparency of Internet health information. In order to avoid falling into the “Tacitus trap”, the government should actively build professional Internet-health-rumor-refuting think tanks and platforms to timely and effectively popularize correct health information. It is essential to take advice from professional scientific research institutions and acquire assistance from news media, so that the Internet health information would be professional, authoritative, and timely. Only a combination of multiple ways can prevent the growth and spread of Internet health rumors to the greatest extent.

### 5.3. From the Perspective of Receiver: Improving Literacy of Health Information of the Public

Improving literacy of health information of the public can enhance their immunity to Internet health rumors, so that it would fundamentally reduce the credibility and block dissemination of Internet health rumors.

Especially, it is necessary to enhance the scientific communication for vulnerable groups, such as children, women, and the elderly. Additionally, scientific communication should specifically aim at the key health issues, that is, food safety, environment issues, and diseases.

In addition, “minority” opinions of Internet health information communication should also be taken into account. The spiral theory of silence holds that when people’s opinions belong to “minority” opinions in the public opinion, they tend to keep silent to avoid isolation. In the field of health information communication, opinions of “minorities”, especially those of professionals, cannot be ignored. How to make professional Internet health information become “dominant opinions” is also a problem worth further consideration.

## 6. Conclusions

By analyzing 50 text materials of Internet health rumors based on grounded theory, three Core Concepts are abstracted, that is, Correlation, Credibility, and Communication. The internal dynamic mutual assistance mechanism among these three concepts is illustrated. Based on the feature, changes, and trends of the Internet, Lasswell’s 5W communication model needs to be reexamined and expanded. Thus, five elements are expanded to eight elements, namely, rumor mongers, rumor refuters, Internet health rumors, rumor-dispelling information, hot events, media channels, receivers, and effect. The eight elements are combined in different forms. The eight-element communication model is viewed from three perspectives. The relationships among eight elements are explained and illustrated to reveal the complicated process and internal mechanism of the generation and communication of Internet health rumors.

Compared with the linear mechanism of the 5W communication model, this model develops the process of generation and communication of Internet health rumors. In addition, it is the application of the 5W communication model at the micro level. The significance of this study is that cutting off the chain among these eight elements may eliminate or weaken the harm and impact of Internet health rumors. Thus, it provides the practical reference value for the government to control Internet health rumors. This article also proposes three strategies for governing Internet health rumors from three perspectives, that is, rumor mongers and the channels, rumor refuters, and rumor receivers. By governing the Internet health rumors from multiple ways, Internet health rumors could be reduced or blocked timely and effectively in the future.

## Figures and Tables

**Table 1:** The results of open coding.

Number	Primary Concept	Text	Frequency	Examples of Reference Points
A1	Bluff	42	171	Eyeball exercises can reduce shortsightedness from 500 degrees to 100 degrees.
A2	Twisting the truth	41	136	A packet of instant noodles contains 25 kinds of food additives, and it takes 32 days to detoxify after eating a bowl of instant noodles.
A3	Being close to life	37	116	Heavy haze would do harm to lungs of people. Eating blood of pig, blood of duck, and other animal blood can clean the lung.
A4	Fear of cancer	18	74	Radiation from geothermal heating can produce cancer.
A5	Life reminder	24	72	The editor learned that the steamer is so poisonous, so do not steam food by using cold water at home.
A6	Food safety	17	65	There are a lot of Toxoplasma gondii in cucumber. It can only be cured by eating selenium. (This rumor actually is for selling healthcare products containing selenium.)
A7	Cognitive contrast	21	59	Compared with patients who refuse chemotherapy, patients who accept chemotherapy would die faster.
A8	Established fact	23	58	Garlic would produce acrylamide when it is fried at high temperature. Acrylamide is a class of 2A carcinogen that is harmful to human body.
A9	Authority of experts	23	57	Chinese experts warn that geothermal heating radiation may cause intellectual disability or amnesia among children.
A10	Pretending to be surprised	20	52	Wi-Fi! The killer hiding in your bedroom at night!
A11	Authority of agencies	25	51	According to the definition of junk food of the World Health Organization, white rice is junk food!
A12	Creating suspense	16	48	Why can’t China improve the radiation protection level of car body like Japan?
A13	Children’s health	13	45	Geothermal heating is slowly killing your children while bringing you warmth.
A14	Research data	17	44	Research from Spain: 90% of shopping receipts contain bisphenol A, which can produce cancer.
A15	Eye-catching headline	36	37	Children would not grow after taking antibiotics once every 7 days! Parents, please watch them carefully for your children!
A16	Inducing curiosity	15	36	Is there nuclear haze in China? Why is this concept blocked on the Internet? What happened?
A17	Environmental Issue	8	32	Drug-resistant bacterium, “the last antibiotic of mankind”, is found in the haze in Beijing, and we could do nothing with it.
A18	Creating panic	13	32	Haze is a first-class carcinogen! It would block 30 million alveoli in your body a year! It would block a third of alveoli for three years!
A19	Literary rhetoric	14	31	Vegetable oil can produce fatty acids which can harm human body, just like effects that climate change threatens the earth.
A20	Marketing strategy	8	29	Cancer patients are cured miraculously after drinking juice of wheat seedling.
A21	Fabricated research	12	27	After 12 days of experiment, the seeds germinated and thrived in the room without router, while most of the seeds of cress and pea died in the room with router!
A22	Chronic diseases	9	24	The ancient prescription of dredging blood vessels would work no matter how serious it is!
A23	Media reports	12	19	Foreign media reported that Ebola virus evolved into zombie virus.
A24	Fabricating the case for a specific person	9	16	John Di Carlo, a 72-year-old man, suffered from leukemia. He used dandelion to make tea and drink it every day. Only four months later, his condition began to improve.
A25	Women’s health	10	15	The radiation of high-speed railways is heavy. It would do harm to unmarried women. So unmarried women do not take them frequently! (Author: this rumor means that the radiation of high-speed railway may cause infertility of unmarried women.)
A26	Traditional medicine	5	15	Dandelion root can kill 98% cancer cells in 48 h!
A27	Moral kidnapping	10	13	Let’s retweet, my friend! For yourself, for your family, and for your friends!
A28	Professional nouns	3	12	Salmonella, which can cause food poisoning, has been detected in the eggs that have speckles.
A29	Political smear	3	12	Many countries, such as France, Germany, and Japan have developed the technology of reducing radiation. The railway research institution of China also develops this technology, but the Ministry of Railways has no plans to use it.
A30	Authoritative journal	8	11	Environmental Research Journal shows that 90% of receipts use thermal paper containing bisphenol A(BPA), which can lead to hormone-dependent cancer.
A31	Skin beauty	3	9	The secret of the three effects of enzyme beauty, which makes you 10 years younger!
A32	The health of the elderly	8	9	The old lady’s cancer was cured by drinking wheat seedling juice.
A33	Inside information	6	9	Warm reminder from the hospital: The pathogens caused by haze mainly aim at children under 12 years old.
A34	False science communication	5	8	The persistent haze in North China belongs to nuclear smog.
A36	Taking advantage of celebrity	5	7	In the speech, Zhong Nanshan (a famous expert in the area of contagion) quoted the relevant statistics of China Environmental Protection Association: For the children with leukemia, 90% of them have had luxurious decoration in their homes. About 2.1 million children die from luxurious decoration every year.
A37	Fawning on foreigners	2	7	Crayfish is a kind of insect that is used to process corpses, and foreigners never eat this food.
A38	Fear of AIDS	1	7	Hong Kong University’s new drug to eliminate AIDS is simply too powerful.
A39	Traditional culture	2	6	Look at the word “rice”. “Food + anti = rice” (in the context of Chinese Character) means the sequence of eating is wrong!
A40	Spread of the virus	1	5	Transgenic mosquitoes may be the chief cause of the outbreak of Zika virus.
A41	Oral health	1	5	It is the correct brushing posture to brush toothpaste directly without touching water.
A42	Historical event	3	5	Japanese biochemical troops used crayfish to dispose of a large number of corpses.
A43	Video commentary	4	5	The photographer said: “This is Toxoplasma gondii (a kind of parasite). It can’t be killed even when the temperature is at 260 °C. It must be cured with selenium.”
A44	Drug safety	2	4	Red alert: Statins may accelerate the aging process.
A45	Men’s health	4	4	Geothermal heating affects the human reproductive system, which is mainly manifested by the decrease of male sperm quality.
A46	Mental health	3	3	Stan said: “If you eat too much corn oil or sunflower oil, the lack of Omega-3 may lead to mental health or aphasia.”
A47	Policy suggestion	3	3	China should formulate a unified national standard for safety of geothermal heating radiation as soon as possible.
A48	False theory	2	3	Cut 2–3 slices of lemon and put it in the hot water, which will turn into alkaline water. It is good to drink it every day.
A49	Educational video	3	3	The video shows that the workers put the plastic into the machine and the machine produces the white particles. The worker also put these white particles into a woven bag originally used for containing rice. (This rumor let people believe that the white particles are the white rice and they would be sold on the market)
A50	Occupational health	1	2	1 million lives have gone per year because of safety accidents of the coal industry in China
A51	Place of incident	2	2	Hookworm has appeared in pork in Zhejiang province, Guangdong province, Hunan province, and other places. The sick pork comes from Guangxi province.
A52	Mathematical formula	1	2	Shrimp + vitamin C = food poisoning
A53	Rumor’s derivatives	1	2	See the article “See how western doctors ‘starve’ cancer cells and cure their cancer”.
A54	The health of the disabled	1	1	People who do not have a sexual partner or have no children can be classified as disabled.
A55	Pretended questioning	1	1	A user majoring in chemistry also questioned that the heat resistance of polyester material used to make beverage bottles is 70 °C, but “the temperature in cars cannot reach 70 °C”!

**Table 2:** Primary Concept and Category Concept.

No.	Category Concept	Primary Concept	Frequency	Connotation
1	Attracting the attention of the public	Eye-catching headline	424	“Attracting public attention” plays a critical role in promoting the generation and communication of Internet health rumors, with the eight primary concepts, e.g., “eye-catching headline” and “pretending to be surprised”, being the important means of “attracting public attention”.
		Pretending to be surprised		
		Media reports		
		Taking advantage of celebrity		
		Cognitive contrast		
		Literary rhetoric		
		Bluff		
		Creating suspense		
2	Faking real information	Traditional culture	286	With real information in ten aspects mixed with fake information, the public finds it difficult to distinguish the genuine from fake. There is a process relationship between “faking real information” and “attracting public attention”; that is, Internet health rumors generally attract public attention first, which then makes it hard for the public to distinguish the faked real information, thereby interfering with the cognition and judgment of the public.
		Traditional medicine		
		Established fact		
		Research data		
		Fabricated research		
		Historical event		
		Authority of agencies		
		Authoritative journal		
		Authority of experts		
		Professional nouns		
3	Fuzzy truth	Being close to life	264	The fuzzy truth in the field of Internet health rumors has the following features: being close to life, twisting the truth, false science communication, false theory, and pretending to raise questions. As a result, the public is unable to clearly recognize the truth about health and will be constantly trapped by the “cage” of Internet health rumors.
		Twisting the truth		
		False science communication		
		False theory		
		Pretending to raise questions		
4	Meeting psychology of the public	Fear of cancer	240	Internet health rumors meet the multiple psychological needs of the public. Fear of cancer and fear of AIDS reveal the public’s psychology of avoiding diseases; life reminder reflects the altruistic psychology; political smear and fawning on foreigners meet the psychology of harm the society (rumors could be taken advantage by people who have malicious intentions).
		Inducing curiosity		
		Fear of AIDS		
		Creating panic		
		Life reminder		
		Political smear		
		Fawning on foreigners		
5	Covering different areas of the health issues	Spread of the virus	147	The need of health information is a significant factor leading to Internet health rumors. Based on the collected materials, Internet health rumors cover eight domains such as spread of the virus, environmental issue, chronic diseases, food safety and drug safety. Therefore, there is nowhere for the public to hide from Internet health rumors while satisfying their needs of obtaining health information.
		Environmental issue		
		Oral health		
		Chronic diseases		
		Skin beauty		
		Mental health		
		Drug safety		
		Food safety		
6	Focusing on the health of different groups of people	Children’s health	76	Focusing on the health of different groups of people promotes the public to rapidly propagate Internet health rumors. The children’s health, women’s health, the health of the elderly, men’s health, occupational health, and the health of the disabled involved in this category have covered almost all groups of people. It strengthens the correlation between receivers and Internet health rumors, which leads the public to actively propagate the rumors by connecting the false information with people around them.
		Women’s health		
		The Health of the elderly		
		Men’s health		
		Occupational health		
		The Health of the disabled		
7	Inducing secondary transmission	Moral kidnapping	49	Intimidating and seducing the receivers to carry on the secondary dissemination passively is the remedy in the case of ineffective active spread of Internet health rumors. Due to moral kidnapping, the receivers have no choice but to spread the rumors. Slogans such as “retweet, bro” in marketing strategy and video commentary are very incendiary. Rumor’s derivatives refer to the mutual assistance between two or more rumors in spreading.
		Video commentary		
		Rumor’s derivatives		
		Marketing strategy		
8	Fictitious information	Fabricating the case for a specific person	21	Those who spread Internet health rumors are good at making up relevant information. Changing the case for a specific person or place of the incident related to health information can often bring Internet health rumors back to life. Policy suggestion makes rumors forward-looking, making it impossible for the receivers to judge whether they are true or false.
		Place of the incident		
		Policy suggestion		
9	Claiming as reliable information	Inside information	14	Internet health rumors often claim to be reliable. To prove themselves reliable, Internet health rumors often resort to inside information, mathematical formulas, and educational videos.
		Mathematical formula		
		Educational video		

**Table 3:** Category Concept and Core Concept.

Core Concept	Category Concept
Correlation	Fictitious information
	Focusing on the health of different groups of people
	Covering different areas of the health issues
Credibility	Claiming as reliable information
	Faking real information
	Fuzzy truth
Communication	Inducing secondary transmission
	Meeting psychology of the public
	Attracting the attention of the public

**Table 4:** Interaction of correlation, credibility, and communication based on 50 Internet health rumors.

		Communication		
		Meeting Psychology of the Public	Inducing Secondary Transmission	Attracting the Attention of the Public
**Correlation**	Fictitious information	6	3	8
	Focusing on the health of different groups of people	16	5	17
	Covering different areas of the health issues	25	10	30
**Credibility**	Claiming as reliable information	0	0	0
	Fuzzy truth	35	11	43
	Faking real information	26	7	32

**Table 5:** Eight-element communication model for Internet health rumors.

Who	What		In Which Channel	To Whom	With What Effect
1 Rumor mongers (communicators)	3 Internet health rumors	5 Hot events—Including various health issues, such as environment, psychology, disease, drug, and food.	6 Media Channel— Website, post bar, BBS, Weibo, blog, WeChat, instant messaging, e-mail etc.	7 Receivers—All groups of people, e.g., the elderly, children, women, and men.	8 Effect—read (not read), believe (not believe), and spread (not spread).
2 Rumor refuters	4 Rumor-dispelling information				

## Appendix A

**Table A1:** Information of 50 Internet health rumors.

No.	Year	Rumor	The Title of News	Platform	Website Address
1	2014	The radiation of Wi-Fi would influence the health of the human body (based on a fabricated experiment)	the Top 10 Rumors for Science of 2014	China Association for Science and Technology (a big national association for researchers, which is financially supported by the Chinese government. It has branches in almost every province and big city in China. One of its goals is for science communication)	https://baike.baidu.com/reference/16532553/4de4MAgwjp6_027xghfhwmNkWpn6kMaMDm1MZtEjO6HnTr2Rbn77tzXYVjbRzwHrJCtp7QYPhBfyVDFGHox-vJfXYk3nG-gbLnjp6xA3 (accessed on 26 October 2022)
2	2014	It would take 32 days to detoxify after eating a bowl of instant noodles	the Top 10 Rumors for Science of 2014	China Association for Science and Technology	https://baike.baidu.com/reference/16532553/4de4MAgwjp6_027xghfhwmNkWpn6kMaMDm1MZtEjO6HnTr2Rbn77tzXYVjbRzwHrJCtp7QYPhBfyVDFGHox-vJfXYk3nG-gbLnjp6xA3 (accessed on 26 October 2022)
3	2014	There are 6 hazards for the radiation from geothermal heating	the Top 10 Rumors for Science of 2014	China Association for Science and Technology	https://baike.baidu.com/reference/16532553/4de4MAgwjp6_027xghfhwmNkWpn6kMaMDm1MZtEjO6HnTr2Rbn77tzXYVjbRzwHrJCtp7QYPhBfyVDFGHox-vJfXYk3nG-gbLnjp6xA3 (accessed on 26 October 2022)
4	2014	Children would not grow after taking antibiotics once every 7 days	the Top 10 Rumors for Science of 2014	China Association for Science and Technology	https://baike.baidu.com/reference/16532553/4de4MAgwjp6_027xghfhwmNkWpn6kMaMDm1MZtEjO6HnTr2Rbn77tzXYVjbRzwHrJCtp7QYPhBfyVDFGHox-vJfXYk3nG-gbLnjp6xA3 (accessed on 26 October 2022)
5	2014	French scientists have proved that transgenic corn would produce cancer	the Top 10 Rumors for Science of 2014	China Association for Science and Technology	https://baike.baidu.com/reference/16532553/4de4MAgwjp6_027xghfhwmNkWpn6kMaMDm1MZtEjO6HnTr2Rbn77tzXYVjbRzwHrJCtp7QYPhBfyVDFGHox-vJfXYk3nG-gbLnjp6xA3 (accessed on 26 October 2022)
6	2014	People who are infected with Ebola virus would become zombies	the Top 10 Rumors for Science of 2014	China Association for Science and Technology	https://baike.baidu.com/reference/16532553/4de4MAgwjp6_027xghfhwmNkWpn6kMaMDm1MZtEjO6HnTr2Rbn77tzXYVjbRzwHrJCtp7QYPhBfyVDFGHox-vJfXYk3nG-gbLnjp6xA3 (accessed on 26 October 2022)
7	2014	Bottled water is poisonous after it is heated by sun	the Top 10 Rumors for Science of 2014	China Association for Science and Technology	https://baike.baidu.com/reference/16532553/4de4MAgwjp6_027xghfhwmNkWpn6kMaMDm1MZtEjO6HnTr2Rbn77tzXYVjbRzwHrJCtp7QYPhBfyVDFGHox-vJfXYk3nG-gbLnjp6xA3 (accessed on 26 October 2022)
8	2019	The radiation of high-speed railways would cause infertility of unmarried women	the Top 10 Rumors for Science of 2019	China Association for Science and Technology	https://m.thepaper.cn/baijiahao_5386755 (accessed on 26 October 2022)
9	2019	Eyeball exercises would cure shortsightedness	the Top 10 Rumors for Science of 2019	China Association for Science and Technology	https://m.thepaper.cn/baijiahao_5386755 (accessed on 26 October 2022)
10	2015	Eating pork blood, duck blood, and other animal blood can eliminate haze	the Top 10 Rumors on WeChat of 2015	WeChat (the most widely used social media in China)	http://health.people.com.cn/n1/2016/0106/c21471-28020014.html (accessed on 26 October 2022)
11	2015	Milk products for children contain specific kind of bacteria, which could cause leukemia	the Top 10 Rumors on WeChat of 2015	WeChat	http://health.people.com.cn/n1/2016/0106/c21471-28020014.html (accessed on 26 October 2022)
12	2015	Drinking the juice of wheat seedling would cure cancer	the Top 10 Rumors on WeChat of 2015	WeChat	http://health.people.com.cn/n1/2016/0106/c21471-28020014.html (accessed on 26 October 2022)
13	2015	Crayfish is used to process corpses	the Top 10 Rumors on WeChat of 2015	WeChat	http://health.people.com.cn/n1/2016/0106/c21471-28020014.html (accessed on 26 October 2022)
14	2015	Using water which contains chlorine for cooking can lead to cancer	the Top 10 Rumors on WeChat of 2015	WeChat	http://health.people.com.cn/n1/2016/0106/c21471-28020014.html (accessed on 26 October 2022)
15	2015	Water used for steaming food actually contains nitrite, which could produce cancer	the Top 10 Rumors on WeChat of 2015	WeChat	http://health.people.com.cn/n1/2016/0106/c21471-28020014.html (accessed on 26 October 2022)
16	2015	Using vegetable oil for cooking would produce cancer	the Top 10 Rumors on WeChat of 2015	WeChat	http://health.people.com.cn/n1/2016/0106/c21471-28020014.html (accessed on 26 October 2022)
17	2015	There are 6 hazards for the radiation of Wi-Fi	the Top 10 Rumors on WeChat of 2015	WeChat	http://health.people.com.cn/n1/2016/0106/c21471-28020014.html (accessed on 26 October 2022)
18	2015	Radiation from geothermal heating would kill your children slowly	the Top 10 Rumors on WeChat of 2015	WeChat	http://health.people.com.cn/n1/2016/0106/c21471-28020014.html (accessed on 26 October 2022)
19	2016	A table showing that some specific kind of food could not be eaten together	the Top 10 Rumors on WeChat of 2016	WeChat	http://www.chinatt315.org.cn/xfjs/2017-1/6/152242.html (accessed on 26 October 2022)
20	2017	Nori and rice are made of plastic	the Top 10 Rumors on WeChat of 2017	WeChat	https://tech.qq.com/a/20180117/034052.htm (accessed on 26 October 2022)
21	2017	Some Chinese medicine or prescription could improve blood flow	the Top 10 Rumors on WeChat of 2017	WeChat	https://tech.qq.com/a/20180117/034052.htm (accessed on 26 October 2022)
22	2018	Drinking hot lemon water can cure cancer	the Top 10 Rumors on WeChat of 2018	WeChat	https://www.sohu.com/a/285836139_120046571 (accessed on 26 October 2022)
23	2019	Salt which contains less sodium would lead to death	the Top 10 Rumors on WeChat of 2019	WeChat	https://news.mydrivers.com/1/664/664851.htm (accessed on 26 October 2022)
24	2019	Pork sold in the market has hookworm in several provinces	the Top 10 Rumors on WeChat of 2019	WeChat	https://news.mydrivers.com/1/664/664851.htm (accessed on 26 October 2022)
25	2016	Drug-resistant bacteria is found in the haze in Beijing	the Top 10 Rumors for Haze of 2016	Ministry of Environment protection of China	https://www.bjnews.com.cn/health/2017/01/03/429287.html (accessed on 26 October 2022)
26	2016	The heavy haze in North China is caused by nuclear pollution	the Top 10 Rumors for Haze of 2016	Ministry of Environment protection of China	https://www.bjnews.com.cn/health/2017/01/03/429287.html (accessed on 26 October 2022)
27	2016	The particles of haze would influence the health of alveoli in human body	the Top 10 Rumors for Haze of 2016	Ministry of Environment protection of China	https://www.bjnews.com.cn/health/2017/01/03/429287.html (accessed on 26 October 2022)
28	2016	Survival rate of patients who accept chemotherapy is only 2.3%	the Top 10 Rumors for Health of 2016	Beijing News (a widely used news media in China)	https://www.bjnews.com.cn/health/2017/01/03/429287.html (accessed on 26 October 2022)
29	2016	People who have no children would be seemed as the disabled	the Top 10 Rumors for Health of 2016	Beijing News	https://www.bjnews.com.cn/health/2017/01/03/429287.html (accessed on 26 October 2022)
30	2016	2.1 million children die from luxurious decoration every year	the Top 10 Rumors for Health of 2016	Beijing News	https://www.bjnews.com.cn/health/2017/01/03/429287.html (accessed on 26 October 2022)
31	2016	Statins (a kind of medicine) may accelerate the aging process	the Top 10 Rumors for Health of 2016	Beijing News	https://www.bjnews.com.cn/health/2017/01/03/429287.html (accessed on 26 October 2022)
32	2016	The pathogens caused by haze mainly aim at children under 12 years old	the Top 10 Rumors for Health of 2016	Beijing News	https://www.bjnews.com.cn/health/2017/01/03/429287.html (accessed on 26 October 2022)
33	2016	Washing hair first when taking showers could cause disease	the Top 10 Rumors for Health of 2016	Beijing News	https://www.bjnews.com.cn/health/2017/01/03/429287.html (accessed on 26 October 2022)
34	2016	Transgenic mosquitoes are main cause of the outbreak of Zika virus	the Top 10 Rumors for Health of 2016	Beijing News	https://www.bjnews.com.cn/health/2017/01/03/429287.html (accessed on 26 October 2022)
35	2016	Using pencil for a long time would cause lead poisoning	the Top 10 Rumors for Health of 2016	Beijing News	https://www.bjnews.com.cn/health/2017/01/03/429287.html (accessed on 26 October 2022)
36	2017	Dipping water on toothbrush before brushing teeth is necessary	the Top 10 Rumors for Health of 2017	Sina website (a widely used news media in China. It covers almost every aspect of news including health areas)	http://health.sina.com.cn/hc/2018-03-07/doc-ifxipenn6660392.shtml (accessed on 26 October 2022)
37	2017	Enzymes (a kind of product) could beautify the skin	the Top 10 Rumors for Science of 2017	Sina website	http://health.sina.com.cn/hc/2018-03-07/doc-ifxipenn6660392.shtml (accessed on 26 October 2022)
38	2017	Eating durian and milk together can lead to food poisoning	the Top 10 Rumors for Food Safety of 2017	Sina website	http://health.sina.com.cn/hc/2018-03-07/doc-ifxipenn6660392.shtml (accessed on 26 October 2022)
39	2017	Gene detection could predict the talent of children	the Top 10 Rumors for Science of 2017	Sina website	http://health.sina.com.cn/hc/2018-03-07/doc-ifxipenn6660392.shtml (accessed on 26 October 2022)
40	2017	Dandelion can cure cancer	the Top 10 Rumors for Healthcare of 2017	Sina website	http://health.sina.com.cn/hc/2018-03-07/doc-ifxipenn6660392.shtml (accessed on 26 October 2022)
41	2018	Shopping receipts contain bisphenol A which can cause cancer	the Top 10 Rumors for Science of 2018	China Science Communication (China Science Communication. It is a platform specifically for science communication)	http://kpzg.people.com.cn/n1/2019/0116/c404214-30551221.html (accessed on 26 October 2022)
42	2018	People have two constitutions, acid or alkaline	the Top 10 Rumors for Science of 2018	China Science Communication	http://kpzg.people.com.cn/n1/2019/0116/c404214-30551221.html (accessed on 26 October 2022)
43	2018	USA Food and Drug Administration has approved a broad-spectrum antibiotic, which has 75% cure rate	the Top 10 Rumor of 2018	Guokr website	https://www.bilibili.com/read/cv1769532 (accessed on 26 October 2022)
44	2018	University of Hong Kong invented a new drug which can cure cancer	the Top 10 Rumors of 2018	Guokr website (a widely used website specifically for science education)	https://www.bilibili.com/read/cv1769532 (accessed on 26 October 2022)
45	2018	Frying Garlic would produce the substance which could cause cancer	the Top 10 Rumors for Healthcare of 2018	Toutiao website (a widely used news media in China)	https://www.sohu.com/a/286786229_120050866 (accessed on 26 October 2022)
46	2018	Coffee from Starbucks could cause cancer	the Top 10 Rumors for Healthcare of 2018	Toutiao website	https://www.sohu.com/a/286786229_120050866 (accessed on 26 October 2022)
47	2018	According to WHO, white rice is junk food	the Top 10 Rumors for Healthcare of Second Quarter of 2018	Toutiao website	https://www.sohu.com/a/240018276_331220 (accessed on 26 October 2022)
48	2018	Cucumber contains a lot of worms	the Top 10 Rumors for Healthcare of Second Quarter of 2018	Toutiao website	https://www.sohu.com/a/240018276_331220 (accessed on 26 October 2022)
49	2018	Eggs which have speckles could lead to food poisoning	the Top 10 Rumors for Healthcare of Second Quarter of 2018	Toutiao website	https://www.sohu.com/a/240018276_331220 (accessed on 26 October 2022)
50	2019	Drinking the tea made by Ginkgo leaf could decrease blood pressure	the Top 3 Rumors for Healthcare of 2019	Shanghai Rumor-Refuting Platform (a platform for refuting rumors in Shanghai)	http://piyao.jfdaily.com:1801/py_4vMWkj9Fy8LJp8MpVOEV/rYZvFcdECbhrPRMUuRm/t6ViQR402s+94+8DZfygLrK9jkaAigp/GLs2ECG9wcD (accessed on 26 October 2022)
